# Comment on Saber et al. (2019), “Commentary: the chronic inhalation study in rats for assessing lung cancer risk may be better than its reputation”

**DOI:** 10.1186/s12989-020-00365-z

**Published:** 2020-07-16

**Authors:** Kevin E. Driscoll, Paul A. Borm, Ishrat Chaudhuri, Len Levy, Mei Yong, David Warheit, Robert McCunney, Günter Oberdörster

**Affiliations:** 1 Ernest Mario School of Pharmacy, Rutgers, New Brunswick, NY 07046 USA; 2 Nanoconsult, Meerssen, Netherlands; 3 Düsseldorf University, Düsseldorf, Germany; 4 Cabot Corporation, Billerica, MA USA; 5 Cranfield University, Cranfield, UK; 6 MY EpiConsulting, Düsseldorf, Germany; 7 Warheit Scientific LLC, Wilmington, Delaware USA; 8 Pulmonary Division, Brigham and Women’s Hospital, Harvard Medical School, Cambridge, MA USA; 9 School of Medicine and Dentistry, University of Rochester, Rochester, NY USA

## Abstract

In their Commentary Saber et al. (Part Fibre Toxicol 16: 44, 2019) argue that chronic inhalation studies in rats can be used for assessing the lung cancer risk of insoluble nanomaterials. The authors make several significant errors in their interpretation and representation of the underlying science. In this Letter to the Editor we discuss these inaccuracies to correct the scientific record. When the science is recounted accurately it does not support Saber et al’s statements and conclusions.

In the Commentary by Saber et al. [1] the authors argue that “the chronic inhalation study in rats can be used for assessing the lung cancer risk of insoluble nanomaterials”. Unfortunately, the Commentary suffers from several major misinterpretations and misrepresentations of the science. When the science is considered accurately it does not support the author’s statements and conclusions. Here we discuss a number of these inaccuracies in order to correct the scientific record.

1. A key position taken by Saber et al. [1] is that “inhalation of the insoluble, low toxicity particles induces lung cancer in rats in the absence of impaired clearance”. To support their assertion, Saber et al. cite reports by Mauderly et al. [2] and Heinrich et al. [3] which summarize results of chronic inhalation studies in rats. However, a review of these reports reveals the findings directly conflict with Saber et al’s statements. Specifically, in the Mauderly et al. and Heinrich et al. studies, all particle exposures associated with lung cancer in rats also produced a marked impairment of lung particle clearance (See Mauderly et al. [2] Fig. 24 and Heinrich et al. [3] Table 9). Moreover, the findings of Mauderly et al. and Heinrich et al. are in agreement with other studies showing that development of lung cancer in rats after inhalation of poorly soluble low toxicity particles (PSLT) occurs only under conditions of excessive lung particle overload [4, 5]. Therefore, the conclusion by Saber et al. [1] that lung cancer occurs in the absence of impaired clearance is not supported by data for PSLT.

2. The authors’ statements on species differences in lung particle clearance after PSLT exposure are incorrect. For example, they write in rats, but not in hamsters or mice, impaired clearance has been observed at high lung burden” and “particle clearance rates in mice, hamsters and rats depend on the lung burden: lower clearance rates are observed with increasing lung burden, but the impaired clearance is only observed in rats”. To support their statements, Saber et al [1] cite a study by Elder et al. [6]; however, the actual results of the Elder et al. study directly contradict the authors’ assertions. Specifically, Elder et al. observed a significant, dose-dependent impairment of lung particle clearance in rats, mice and hamsters exposed for 13 weeks by inhalation to carbon black (CB) (see Elder et al. [6] Table 4) and concluded all three species showed lung particle overload under similar exposure scenarios. The Elder et al. findings concur with other studies, including Muhle et al. [7] who demonstrated that rats and hamsters both exhibit impaired lung particle clearance at high lung burdens of PSLT.

3. To support their contention that rat lung cancer after PSLT inhalation reflect a human hazard, the authors discuss coal miner epidemiology as an example of “occupational exposure to carbon dust”. The basis of this argument is seriously flawed as coal miners are exposed to a complex mixture of substances which can include soluble materials as well as crystalline silica, a known human carcinogen [8]. As such, coal miner exposure is not a surrogate for exposure to a PSLT and is much more than simply exposure to carbon dust. Having not appreciated the compositional differences between mixed dust coal miner exposure and exposure to a PSLT, the authors selectively discuss two coal miner epidemiology studies that reported an apparent increase in lung cancer risk [9, 10] among numerous studies that have reported no excess lung cancer risk [11].

4. Saber et al. [1] argue a comparison of lung cancer risk estimates for diesel exhaust derived from inhalation studies in rats and occupational epidemiology indicates “chronic inhalation studies in rats do not overestimate carcinogenic risks”. As with the coal miner exposures, diesel exhaust exposure involves a complex mixture of carbonaceous particles and adsorbed organics including carcinogenic nitro and aromatic compounds [12]. As such, the diesel exhaust epidemiology is not relevant to PSLT exposures. Considering the epidemiology for actual PSLTs, we note that titanium dioxide (TiO_2_) and CB both have an extensive high-quality data base indicating no exposure-response relationship to lung cancer risk [13, 14]. Le at al [13] reported a meta-analysis of greater than 25,000 TiO_2_ production workers and showed no elevated risks for lung cancer mortality or non-malignant respiratory disease. Also, a meta-regression of CB mortality studies found no exposure-response relationship for CB and lung cancer in upwards of 9000 CB production workers [14].

In summary, understanding the relevance of rodent toxicology studies to human risk assessment is critically important. Unfortunately, in their Commentary, Saber et al. [1] make several significant errors in their interpretation and representation of key studies which, when accurately considered, do not support the author’s statements and conclusions on relationships between lung clearance and rat lung cancer; species differences; and the predictiveness of rat lung cancer for nanosized PSLT. Germane to this discussion are the outcomes of a recent workshop on inhaled PSLT where a panel of 15 experts on PSLT toxicology and regulatory matters reached consensus that *“rat lung tumors occurring with PSLT only under lung particle overload are not relevant to humans under non-overload exposure conditions”* [15].

## Data Availability

Not applicable.
